# Roles of Inorganic Oxide Based HTMs towards Highly Efficient and Long-Term Stable PSC—A Review

**DOI:** 10.3390/nano12173003

**Published:** 2022-08-30

**Authors:** M. Shahinuzzaman, Sanjida Afroz, Hamidreza Mohafez, M. S. Jamal, Mayeen Uddin Khandaker, Abdelmoneim Sulieman, Nissren Tamam, Mohammad Aminul Islam

**Affiliations:** 1 Institute of Fuel Research and Development, Bangladesh Council of Scientific and Industrial Research (BCSIR), Dhaka 1205, Bangladesh; 2 Department of Physics, University of Rajshahi, Rajshahi 6205, Bangladesh; 3 Department of Biomedical Engineering, Faculty of Engineering, Universiti Malaya, Jalan Universiti, Kuala Lumpur 50603, Selangor, Malaysia; 4 Centre for Applied Physics and Radiation Technologies, School of Engineering and Technology, Sunway University, Bandar Sunway 47500, Selangor, Malaysia; 5 Department of General Educational Development, Faculty of Science and Information Technology, Daffodil International University, DIU Rd, Dhaka 1341, Bangladesh; 6 Department of Radiology and Medical Imaging, Prince Sattam bin Abdulaziz University, Alkharj 11942, Saudi Arabia; 7 Department of Physics, College of Sciences, Princess Nourah bint Abdulrahman University, P.O. Box 84428, Riyadh 11671, Saudi Arabia; 8 Department of Electrical Engineering, Faculty of Engineering, Universiti Malaya, Jalan Universiti, Kuala Lumpur 50603, Selangor, Malaysia

**Keywords:** perovskite solar cell, stability, efficiency, inorganic oxide materials, HTM, interface engineering

## Abstract

In just a few years, the efficiency of perovskite-based solar cells (PSCs) has risen to 25.8%, making them competitive with current commercial technology. Due to the inherent advantage of perovskite thin films that can be fabricated using simple solution techniques at low temperatures, PSCs are regarded as one of the most important low-cost and mass-production prospects. The lack of stability, on the other hand, is one of the major barriers to PSC commercialization. The goal of this review is to highlight the most important aspects of recent improvements in PSCs, such as structural modification and fabrication procedures, which have resulted in increased device stability. The role of different types of hole transport layers (HTL) and the evolution of inorganic HTL including their fabrication techniques have been reviewed in detail in this review. We eloquently emphasized the variables that are critical for the successful commercialization of perovskite devices in the final section. To enhance perovskite solar cell commercialization, we also aimed to obtain insight into the operational stability of PSCs, as well as practical information on how to increase their stability through rational materials and device fabrication.

## 1. Introduction

Perovskite, an organic–inorganic hybrid material, tends to be a promising light-harvesting material. In 2009, the Miyasaka group [1] reported a perovskite solar cell (PSC) of 3.8% using a DSSC system configuration with liquid electrolyte based on MAPbI_3_ (MA = CH_3_NH_3_^+^). Park group [2] obtained a nearly doubled power conversion efficiency (PCE) of 6.5% in 2011 using a high concentration perovskite precursor solution. Since then, several efforts have been made to enhance PSC photovoltaic efficiency from various angles, including perovskite layer fabrication methods, interface engineering, cell architecture design, and development of the hole transporting materials (HTM), an electron transporting materials (ETM). A certified PCE of 22.7% has already been achieved via the above optimization [3]. Although the PCE currently available is appealing, the PSCs still have low stability (thermal, light, and moisture stability), which hinders their commercialization.

The discovery of high-efficiency and highly stable perovskite solar cells has sparked extensive research, which is still ongoing [4,5]. Particularly, organometallic semiconducting perovskite has a direct band gap with high absorption coefficients [6] that enables efficient light absorption in ultra-thin films. Furthermore, it has a long diffusion length [7,8,9], low exciton binding energy [10,11], high carrier mobility [12,13], and simple and easy preparation techniques [14] that help to get high efficiency and low-cost showing promising alternative to the conventional crystalline silicon-based solar cell. Moreover, perovskite materials can be implemented in two different cell structures, either as planer (n-i-p) or inverted (p-i-n) architecture. Moreover, both architectures could be (i) regular structures in which no mesoporous layer is employed, and (ii) mesoscopic structures where a mesoporous layer is needed. The significant improvement in efficiency already achieved in all kinds of architecture, and the stability of PSCs remain the key concerns for the researchers at present time. Many changes were made to the working electrode, the electron transport layer (ETL), and the hole transport layer (HTL) to improve their stability and charge transport properties. The hole transporting materials is a very much important factor in PSCs to achieve high efficiency and performance. It acts as the mediator to transfer positive charges (Holes) between the perovskite and counter electrode [15]. Particularly, highest efficiency (PSCs) are achieved with organic HTL such as 2,2,7,7-tetrakis-(N,N-di-pmethoxyphenylamine)-9,90-spiro-biuorene(spiro-MeOTAD) [16]. The other most commonly used organic HTMs are poly(3,4-ethylene dioxythiophene) (PEDOT) or poly(3,4-ethylene dioxythiophene):poly(styrene sulfonate) (PEDOT:PSS) [17], poly-[[9-(1-octylnonyl)-9H-carbazole-2,7-diyl]-2,5-thiophenediyl-2,1,3-benzothiadiazole-4,7-diyl-2,5 thiophenediyl] (PCDTBT) [18,19], poly-[3-hexylthiophene-2,5-diyl] (P3HT) [20,21], 4-(diethylamino)-benzaldehyde diphenylhydrazone (DEH) [22], poly-triarylamine (PTAA) [19,23], N,N-dialkyl perylene diimide (PDI) [24], polypyrrole (PPy), polyaniline (PANI) [25], etc. From a commercial standpoint, the production of solar cells utilizing an organic hole transport layer has encountered numerous challenges, the most significant of which are material cost and stability. Particularly, high purity spiro-OMeTAD is more costly than novel metals such as gold and platinum, which are commonly used as a counter electrodes. Commercially available spiro-OMeTAD is nearly ten times more expensive than platinum and gold. On the other hand, organic HTMs are typically hygroscopic in nature and that’s why it has an impact on the PSCs’ general stability.

In contrast, several low-cost inorganic HTLs were also proposed and implemented for enhancing the stability of PSCs, among them, some of the HTMs are CuSCN [26], NiO_x_ [27], Cu_2_O or CuO [28], CuI [29], CuGaO_3_ [30] and CuAlO_2_ [31], MoO_x_ [32], CuS [33], MoS_2_ [34], and polymer electrolyte [35]. The above-mentioned HTMs have shown potential as they offer suitable properties for application in PSCs including the suitable band-to-band alignment with the perovskite layer, low resistivity, and low-cost solution-process ability [2]. In the case of inorganic HTM, increased demand for inorganic HTM will certainly lower the cost of large-scale manufacturing, while organic HTM will likely stay expensive due to the preparation processes and materials with very high purity required for solar cell applications. These are the primary reason why researchers have concentrated their efforts on the development of an inorganic HTM. Consecutively, the quest for the perfect HTM is a great topic yet. There is a lot of literature on various HTMs, but only a few of them show promise in terms of improving the overall efficiency and stability of the PSCs. Several approaches have evolved to utilize inorganic p-type semiconductor materials, such as NiO_x_, CuO_x_, etc., focusing on developing non-hygroscopic and highly conductive HTMs [36]. Moreover, carbon-based materials, including graphene, activated carbon, carbon black, graphite powder, carbon nanotube (CNT), etc., have been employed in the case of HTM-free PSC structures [37,38,39,40]. In particular, current approaches to HTMs are low cost, high mobility, low absorption in the visible region, ease of synthesis, and good chemical stability that could ensure high efficiency and stable PSCs. In recent years, several review works have been published on inorganic metal oxide hole-transporting materials for perovskite solar cells in different formats and among them, some are focused on fabrication way, some are on efficiency and some are focused on stability. A list of some important review articles on inorganic metal oxide-based PSCs for the year 2015–2021 is shown in Table 1. However, in this review, we are summarizing different types of inorganic HTMs which have been employed in the fabrication of PSCs focusing on their impact on device efficiency and stability and we have gathered the information for the last ten years till now. This work focuses on the all necessary concerns for effective inorganic HTM-based PSCs such as device structure, fabrication technique, efficiency, and stability.

## 2. Perovskite Solar Cell

PSCs (organic-inorganic perovskite solar cells) are considered a significant recent breakthrough in photovoltaics and have recently received great attention [49]. The power conversion efficiency (PCE) of PSCs has already enhanced from 3.8 percent to 25.8 percent through the system engineering and materials design regarding the correct optoelectronic aspects in just 10 years [56]. Thus, PSCs are recognized as the best alternative approach for replacing the costly and market-dominant crystalline silicon solar cells [51,57,58,59,60]. Moreover, PSCs are more cost-effective than conventional inorganic semiconductor thin-film solar cells, such as CIGS and CdTe [52]. The real obstacle to commercialization, however, is maintaining long-term stability. PSCs are particularly susceptible to deterioration when exposed to moisture, oxygen, heat, and light, and they must address before they can use in practical applications. Perovskite is itself very reactive due to the presence of vacancies in its structure. This is the defect of perovskite and it can encourage ion migration through the perovskite layer. Furthermore, the organic cations which are used in PSCs are hygroscopic in nature. When the PSCs are contacted with moisture, the water molecule reacts with it and forms a weak hydrogen bond with the cation which results in the formation of a hydrated perovskite phase [52]. Oxygen, heat, and UV influence this chemical reaction and favor the instability of PSCs. For commercialization, PSCs must be able to operate without major degradation for almost 25 years in outdoor conditions [61]. PSCs have so far been claimed to have one-year stability, which is considerably less than the PV systems that are already on the market. Thus, it is evident that the stability and limited longevity of PSC PV are the main factors impeding its commercialization [62].

**Figure 1:**
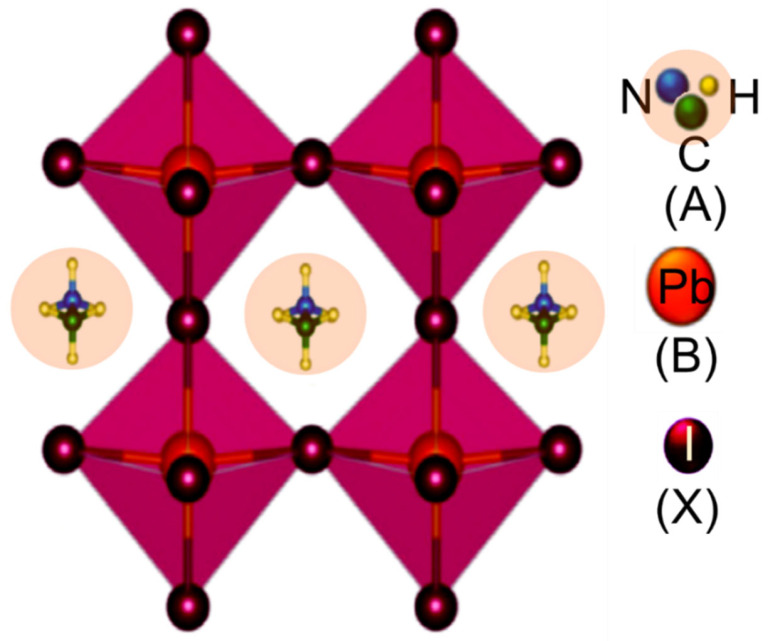
Crystal structure of perovskite with a general chemical formula of ABX_3_ (in the case of CH_3_NH_3_PbI_3_, A represents the CH_3_NH_3_, B represents the Pb, and X represents I).

The basic building block of the perovskite structure, ABX_3_, is shown in Figure 1, where A and B are cations with different sizes (A being larger than B) and X is an anion [63]. Figure 1 represents the simplest structure made up of cubic symmetry of corner-sharing BX_6_ octahedra, where the B cations are in the middle of the octahedron and the X anions are at the corners [64,65]. In the gap of cuboctahedra, the A cations are located at interstices, surrounded by eight octahedral, and form a cubic Pm_3_m crystal structure [66]. In the case of frequently used perovskites in solar cells are organo-metal halide perovskite materials, where ‘A’ may be an organic or inorganic cation (i.e., MA^+^, FA^+^, Cs^+^, K^+,^ and Rb^+^), while ‘B’ is a metal cation (i.e., Pb^2+^ or Sn^2+^), and ‘X’ is a halide anion (i.e., Cl, Br, I, etc.) [67,68].

It should be mentioned that the A, B, and X ions must satisfy this formula, t = (RA + RX)/2 (RB + RX), where RA, RB, and RX are the corresponding ionic radii and t = 1, is the tolerance factor. For most cubic perovskite structures, 0.8 t 0.9 is found quantitatively. In the case of lower symmetry, the value of “t” is very small and then the film structure will be tetragonal or orthorhombic. Alternatively, if t ≥ 1, hexagonal structures are formed, and layers of face-sharing octahedra are added to the structure [67,68]. Moreover, organometal halide perovskites have already been proven several outstanding optoelectronic properties, such as a large absorption coefficient, direct bandgap, small exciton-binding energy, ambipolar semiconducting characteristics, long charge-carrier diffusion length with high charge-carrier mobility [67,68]. Furthermore, the researcher proposed hybrid organometal perovskite material with structure ABX_3−x_Y_x_, for example, MAPbI_3−x_Cl_x_ and MAPbI_3−x_Br_x_, which has tunable optical properties. The tunable optical properties make it easier to experiment with device performance and improve PSCs’ overall performance [68]. On the other hand, perovskite films can be prepared by versatile low cost and simple film deposition methods, such as spin-coating [69,70], sequential deposition [71,72], and evaporation [73,74] techniques. Low-temperature spin coating is the simplest method to fabricate low-cost and high-efficiency PVSC devices. However, it is very challenging to form continuous perovskite films means non-fully covered perovskite films by spin-coating via the direct methyl ammonium halide and lead iodide (PbI_2_) mixed precursor solution [75,76]. All the above process has their own limitation and commercial viability.

**Figure 2:**
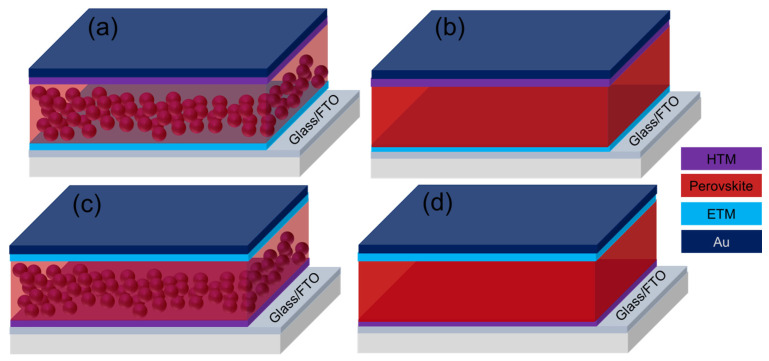
Device architectures of perovskite solar cells; (**a**) normal mesoscopic, (**b**) normal planar, (**c**) inverted mesoscopic, and (**d**) inverted planar structure.

Miyasaka and co-workers first reported the liquid-electrolyte-based dye-sensitized solar cells (DSCs) of PCE as a maximum of 3.8% using MAPbI_3_ and MAPbBr_3_ perovskites as light absorbers [1]. However, due to the dissolution of the perovskites in the liquid electrolyte, the system was found to be very unstable. In 2012, a significant advance was made independently by Grätzel et al. [60] and Snaith et al. [77] where the liquid electrolyte was replaced with a small-molecule-based hole-transporting material (HTM), 2,2′,7,7′-tetrakis(N,N-di-p methoxyphenylamine)-9,9′-spirobifluorene(spiro-OMeTAD). The perovskite is penetrated the mesoporous TiO_2_ (mp-TiO_2_) scaffold with an additional capping layer as shown in Figure 2a, which is covered with a thin layer of the HTM in a typical mesoscopic PSC. Finally, a metal electrode, preferably gold (Au), is deposited on the top of the HTM [61,77]. Instead of TiO_2_, Al_2_O_3_ insulating scaffold can also be used in this mesoscopic structure [77]. The device has been found to work well, signifying that the perovskite could serve as a light harvester as well as an electron transporter (ETM). This finding led to a planar PSC configuration without the mesoporous scaffold as shown in Figure 2b. Particularly, in planar PSCs, the perovskite is simply sandwiched between a thin layer of HTM and a compact ETM, such as TiO_2_, ZnO, SnO_2_, etc. [78]. Moreover, HTM-free PSC also reported where the perovskite works as a hole transporter as well as a light absorber [79]. Moreover, ambipolar semiconducting characteristics of the perovskite support fabricating PSC in an inverted fashion, which is typically known as inverted PSCs. Figure 2c represents the mesoscopic inverted PSCs where a p-type mesoporous matrix (such as NiO) is used to deposit the perovskite, and then, a thin layer of ETM is deposited on top of the perovskite [80]. Finally, fabrication has been completed by depositing a metal electrode, such as silver (Ag), by the thermal evaporation technique. Analogous to usual architectures, the PSCs in inverted structure can be fabricated as shown in Figure 2d, where the perovskite layer is sandwiched by an ETM, such as PCBM, and a thin HTM, such as poly(3,4-ethylene dioxythiophene):poly(styrene sulfonic acid) (PEDOT:PSS) [54].

**Figure 3:**
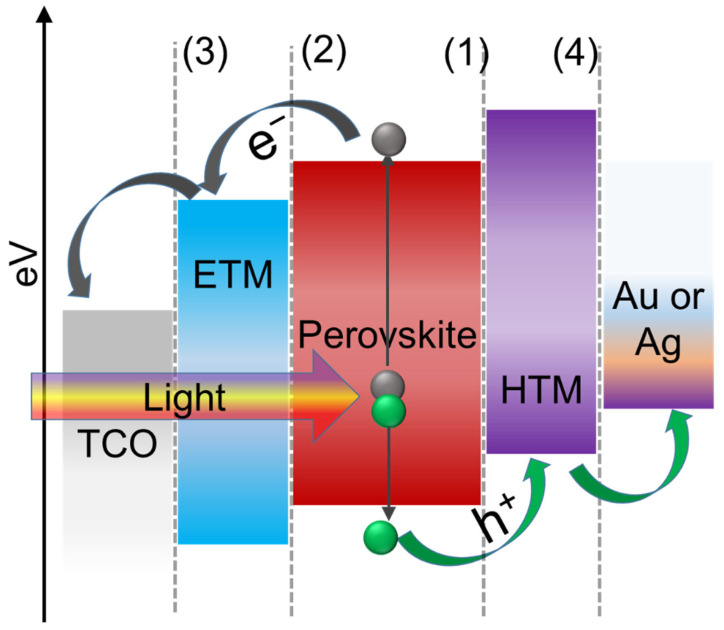
Energy level diagram and the carrier transport mechanism of perovskite solar cell in normal configuration (Interfaces in planar PSCs showing (1) HTL/perovskite interface, (2) perovskite/ETL interface, (3) ETL/cathode interface, and (4) HTL/anode interface).

As previously mentioned, PSCs use primarily two types of system structures (normal and inverted) and obviously, transparent conductive oxide (TCO) (such as ITO or FTO), HTM, perovskite layer, ETM, and contact electrodes (like Au and Ag) are the main components of both structures as shown in Figure 2a,b. The energy band diagram of a normal configuration, shown schematically in Figure 3, depicts the transporting trajectory of electrons and holes during the action. Excitons are produced and then separated into free carriers when sunlight illuminates the perovskite active layer. The generated electrons and holes can then be transported to each interface and injected into ETM and HTM, respectively. Finally, counter electrodes capture electrons and holes in ETM and HTM, respectively, transport them to an external circuit, and generate current [55,81]. Charge separation between MAPbI_3_ and HTM such as spiro-MeOTAD was observed in transient absorption spectroscopy, but electron injection at open-circuit conditions was not detected yet [61]. It has already been confirmed that HTM plays a crucial role in carrier separation and transport in PSCs [50] which will be discussed in this study for most of the inorganic HTMs used in PSCs.

Particularly, there are primarily four types of interfaces in the inverted and/or normal structure of PSCs as shown in Figure 3. Each of the interfaces is methodically related to interfacial carrier dynamics including charge separation, charge injection, charge transport, charge collection, and recombination processes, and consequently affects how well the device functions in the end. Charge transport, extraction, and collection in real-time operation of PSCs are usually accompanied by charge recombination, which is closely related to PCE, stability, and hysteresis. It clearly shows that interface engineering is essential for developing effective and reliable PSCs. The functions and significance of interfaces in terms of structure, operational mechanism, interfacial carrier dynamics, and PSC characterization approaches will also be discussed in this review.

## 3. HTM in PSC

**Figure 4:**
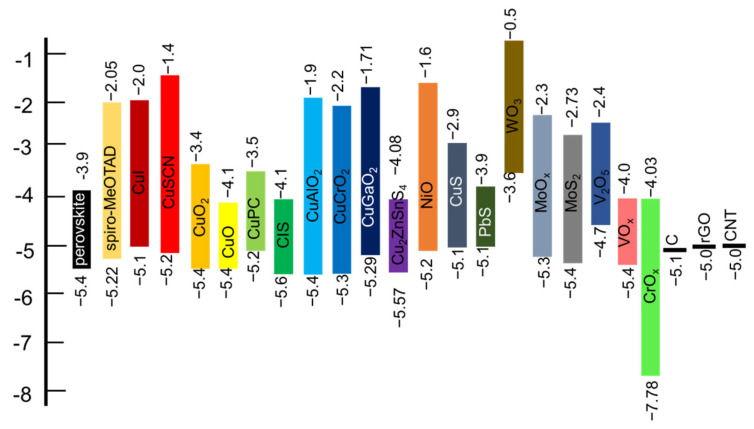
The energy level diagram for IHTMs and other materials used for perovskite solar cells [45,48,50].

In particular, the main function of HTM in PSCs is to collect and transport holes from the perovskite layer. Several prerequisites need to be satisfied by an ideal HTM. First, the hole mobility should be enough high to ensure that holes can be transported to the nearest contact electrode. Secondly, the energy of the valence band maximum (VBM) needs to be higher than that of the perovskite layer. At the same time, to effectively block the movement of electrons towards HTM from perovskite, a higher conduction band minimum (CBM) of HTM than perovskite should be assured. Typically, if electrons move to the HTM, they will recombine with the holes that have in the HTM and increase the carrier recombination loss. Figure 4 shows the band diagram of some representative inorganic HTMs (IHTMs) along with perovskite and spiro-MeOTAD [48,50]. Moreover, for achieving long-term stability, the chemical- and photo-stability of HTM are equally essential. Next, in the case of inorganic HTM deposition via a solution process, solubility in organic solvents and film-forming ability are important. The critical issue is that the organic solvent used to dissolve the inorganic HTM on top of the perovskite layer must be inert to the underlying perovskite film. Finally, to avoid the loss of incident photons, a high optical transmittance is required, particularly in the case of inverted structures. Furthermore, toxicity and cost of the material is also an important concern in the commercial context. Table 1 shows the summary of the price and mobility of the commonly used HTMs in PSCs. Particularly, organic HTM (OHTM) is one of the costliest layers in PSC fabrication as can be clear from Table 2. Even though high PCEs have been achieved using OHTMs yet to date, a significant amount of material is lost during the deposition technique (usually solvent-based process), further raising the final product price. Various types of Inorganic HTMs have been designed for high-performance and stable PSCs in recent years based on the above basic requirements.

## 4. Major Fabrication Techniques for IHTM

### 4.1. Spin Coating

**Figure 5:**
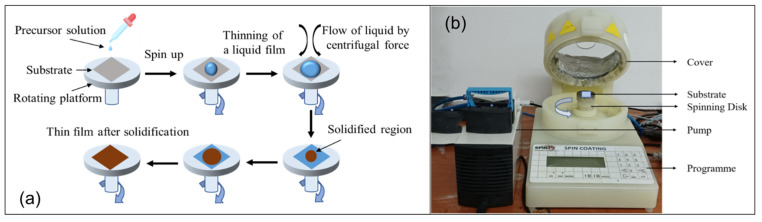
Schematic Image of Spin coating (arrow sign in (**a**) shows the work flow).

Nickel oxide thin films can be synthesized using various techniques. Among them, the spin coating technique is a more convenient and easy way by which homogenous and high-quality films can be obtained at a low cost [90]. This technique is used to deposit a thin layer of materials on a flat surface by spinning the substrate at high speeds ranging from 1000 to 8000 rpm. The deposited solution precipitates and dries the film in this approach by evaporating the solution with high-velocity airflow associated with high-speed rotation. The film thickness is influenced by various factors, including spinning speed, spinning time, solution velocity, solution concentration, vapor pressure, temperature, and moisture [28,91,92]. The key advantages of spin coating are the reduced material loss and the low cost. The difficulty of multilayer deposition, the possibility of contaminants (solvent, oxygen, humidity), and the difficulty of depositing precisely regulated film are all disadvantages of this approach. Previously, many researchers successfully deposited the inorganic metal oxide-based thin films using the spin coating process. Figure 5a shows a work flow of a simple spin coating technique and Figure 5b schematic image of a simple spin coater.

### 4.2. Sputtering

Sputtering is one of the prominent techniques to deposit metal oxide thin film on the perovskite device. To get the uniform metal oxide layer it needed to maintain a high vacuum of <5 × 10^−4^ Pa for the sputtering process. In the case of the DC magnetron sputtering system, the target substrate is negatively biased and, in this case, the substrate is grounded. To create the plasma, the process gas, such as argon, is discharged, accompanied by secondary electron emission. Due to the Ar^+^ bombardment, neutrally charged particles are expelled from the target and deposited on the substrate alongside the sputter target, generating a uniform layer. The Lorentz force confines the movement of electrons by embedding a permanent magnet beneath the target. Due to their much higher mass, Ar^+^ ions are less affected. The DC magnetron is often used for conducting targets. Insulating targets are deposited using RF magnetron sputtering [44].

**Figure 6:**
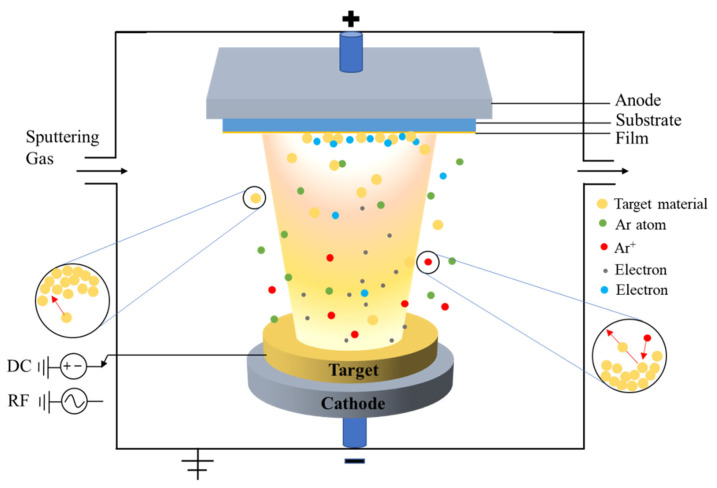
General schematic diagram of an RF-DC magnetron sputtering system.

For example, in the sputtering process of NiO_x_ the Ni metal was used as the target while oxygen was used as the carrier gas at a constant pressure. Using the sputtering method, the thickness and properties of the thin film depend on several parameters. The radio frequency power, sputtering pressure and temperature, and substrate to target distance are some of the main parameters. The main advantages of the sputtering technique are to get uniform and pinhole-free thin film with deposition of a large surface area [93,94]. Figure 6 shows a general schematic diagram of an RF-DC magnetron sputtering system.

### 4.3. Spray Pyrolysis

**Figure 7:**
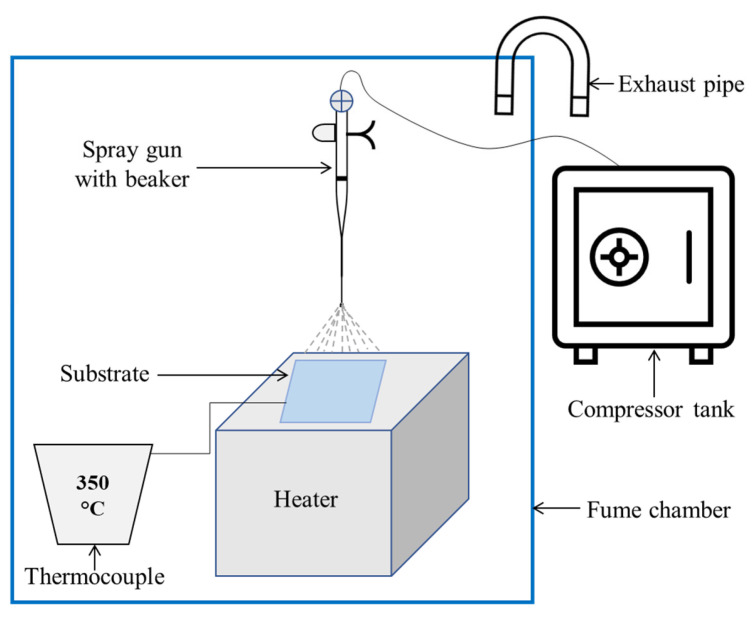
Schematic diagram of the spray coating process.

Spray pyrolysis is a technique where a solution was sprayed on a hot substrate to deposit the targeted metal oxide layer [85]. In this process, the substrate material like FTO is kept on a hotplate at a high temperature (around 500 °C). After drying the substrate, the targeted metal salt solution was sprayed within a certain time by an air nozzle onto the FTO substrate. Afterward, the coated and treated FTO material was then maintained at a high temperature for a while to induce crystallization before being prepared as a transparent film, as shown in Figure 7. For example, the nickel acetylacetonate solution was prepared by adding 0.2 mol/L salts in acetonitrile to deposit NiO_x_ film. The substrate was first heated at 450 °C for 30 min [95]. A spraying head was employed to purge the solution with oxygen gas. A ball valve was used to control the flow rate of the spray, which was then heated to 450 °C for 15 min before being cooled to room temperature.

### 4.4. Solution Combustion Process

**Figure 8:**
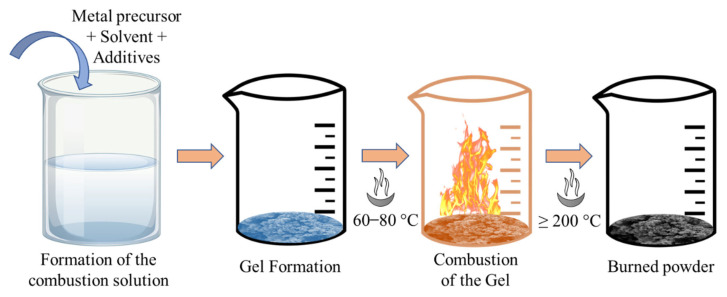
Schematic diagram of the solution combustion process (straight arrow sign shows the work flow).

The solution combustion process is a high-temperature-based self-propagation synthesis technique that is an effective energy-saving synthesis process for varieties of advanced materials. In this process, the reaction solutions are ignited by means of external thermal energy sources at high temperatures. This process is fulfilled with two steps. Firstly, the reaction solutions are in the liquid state and allow to mix of the reactants to form a uniform and precise formation of the required composite in Nanosized at moderate temperature. Then the high purity of the product and the crystallinity achieve utilizing high temperature. Figure 8 shows a schematic diagram of the solution-combustion synthesis process.

The solution combustion process is a good technique by which 20.1% highest efficiency of NiO_x_ with a certain amount of Li and Co as dopant has been achieved by Wang S. et al., (2019) [96]. In this study, 6% of degradation was reported after 30 days in ambient air with 30–40% RH. Recently, another study reported a 19.7% of power conversion efficiency of NiO_x_-based perovskite solar cells [91]. The 18% of degradation with 30% RH was reported at 900 h in ambient air. In this method, combustion-derived Cu:NiO_x_, Ni(NO_3_)_2_·6H_2_O (0.95 mmol), and Cu(NO_3_)_2_·3H_2_O (0.05 mmol) were dissolved in 2-methoxy ethanol (10 mL). The produced solution was stirred for 1 h at 50 °C, then 10 L of acetylacetone was added and stirred for another 1 h. For the combustion and traditional processes, the spin coating was employed to coat Cu:NiOx thin film onto the surface of the ITO-coated substrate, which was subsequently held in the ambient environment for 1 h at 150 °C and 500 °C, respectively. Due to its straightforward synthesis approach for materials, the solution process is generating a lot of interest [97,98].

### 4.5. Other Methods

**Figure 9:**
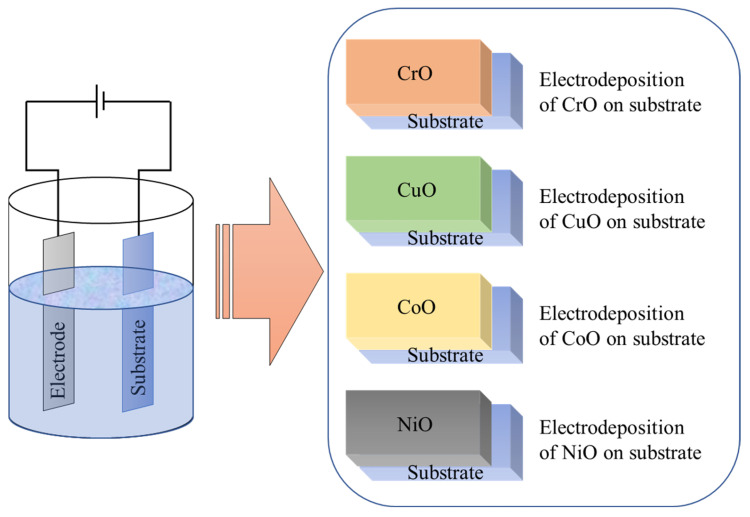
Schematic diagram of electrodeposition technique for IHTL (commonly fabricated materials using electrodeposition is showing in the box).

There have been some other deposition techniques to make the inorganic metal oxide-based perovskite solar cells which reported good power conversion efficiency. The Atomic layer deposition technique is one of the important techniques to deposit NiO_x_ as the HTL. The highest efficiency of 18.5% was achieved by this technique with 13.3% of degradation after 500 h in ambient air under one sun illumination at 85 °C and the highest PowerPoint [99]. Electrodeposition, drop-casting, atomic layer deposition, vacuum deposition, vacuum thermal evaporation, and e-beam evaporation techniques were also used to deposit the metal oxide-based HTL for perovskite solar cells [99,100,101,102]. Figure 9 showed the schematic diagram of the electrodeposition technique for PSC solar cells. Recent studies and advancements of different metal oxide-based perovskite solar cells over the last 7 years are studied and discussed below to find a better direction for future work on inorganic metal oxide-based perovskite solar cells.

## 5. Inorganic Oxide-Based HTM in PSCs

### 5.1. NiO_x_

The NiO_x_ is a very prominent HTM to derive better reliable and stable PVSCs among the other inorganic oxide-based HTMs. It possesses a wide bandgap with a deep-lying valance band which is a well-fitted energy level to provide a high open-circuit voltage (V_OC_) for perovskite material [92]. Among the various inorganic HTMs, NiO_x_ reveals the most promising materials due to its low cost, easy and cost-effective synthesis process, earth-abundant material, and nontoxicity [48]. Furthermore, NiO_x_ is a p-type semiconducting material with a wide range of bandgap, superior transparency, and proper work function with a valance band. Compared with various conventional materials such as PEDOT:PSS and spiro-OMeTAD, NiO_x_ is a more suitable HTM due to its non-acidic and hygroscopic nature. Interestingly, the crystalline NiO_x_ film surface may have a degree of defect which may influence the transfer of charge through the consequent perovskite interface [46]. Interestingly, however, it may also affect the morphology and crystal structure of the perovskite film grown on to the NiO_x_. Thus, the stability and the performance of the NiO_x_-based PVSCs mostly depend on the surface morphology of the NiO_x_ HTL and it is required to improve the surface structures of the NiO_x_ [103,104,105].

Previously, many studies have been conducted to develop the material properties as well as the deposition techniques for NiO_x_-based PSCs [41,106,107,108,109,110,111]. The structural properties of NiO_x_ can be improved by improving the synthesis route. Nowadays, the NiO_x_ nanoparticle and quantum dots gains interest in this field due to their large surface area and uniform crystal structure. Furthermore, the various post-surface treatments, such as UV-ozone treatment and Oxygen plasma, could be applied to develop the interface properties of HTL NiO_x_-based HTL. These treatments can improve the work function which is good for better energy level match and reduction of V_OC_ loss, wettability as well as increase the Ni^3+^ amount which is necessary to increase the Ni vacancies for greater hole conductivity [41]. Various synthesis routs of NiO_x_-based HTLs, such as sol-gel method, spin coating method, chemical bath deposition, chemical vapor deposition, spray pyrolysis, e-beam evaporator, RF magnetron sputtering, electro-deposition process, screen-printing technology, and pulsed laser deposition, have been improved with the highest efficiency of 21.66% [106]. However, there has still a lot of opportunities to develop this prosperous material as the HTM in Perovskite solar cells.

**Figure 10:**
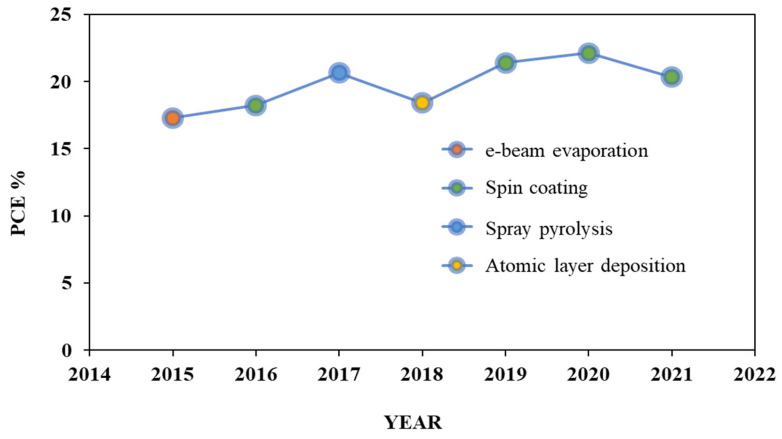
Record of PCE for NiO_x_ HTL-based PSCs using different deposition techniques reported at the recent time.

There are different synthesis and deposition techniques that have been employed to improve the performance and stability of PSC-based oxide HTMs. Among them the most used methods are spin coating, spray pyrolysis, RF sputtering, chemical vapor deposition, thermal evaporation, and e-beam evaporation. However, most of these methods are costly and it is still required to develop the deposition techniques as well as the materials. Table 3 shows the efficiency of NiO_x_-based perovskite solar cells using different deposition techniques. From this study, it has been shown that the spin coating technique is widely used for the deposition of NiO_x_-based HTLs in PVSCs. Figure 10 shows the record of PCE for NiO_x_ HTL-based PSCs from 2015 to 2021 where the year 2020 with the spin coating technique achieved the highest efficiency.

Until now, the highest photo-conversion efficiency of perovskite solar cells using NiO_x_ HTL is 22.13% with the highest FF, V_OC_, and J_OC_ of 0.82, 1.14, and 23.44, respectively [107]. The n-i-p structure of the perovskite device using spin-coated with NiO_x_ as the HTL showed emerging efficiency of 21.66% [106]. In this study, the NiO_x_ bi-functional layer (hole transporter and passivation) was deposited using the spin coating method on the top of the perovskite layer twice. It is also clear that the NiO_x_/Spiro device has higher efficiency than the pure spiro device. In this structure, the stability also increased by preventing the Li-ion diffusion between the spiro and the perovskite layer. Another study showed that the NiO_x_-based hole transporting layer on a perovskite device showed 21.4% power conversion efficiency with an 83.6% of fill factor [108]. Shizhong Yue et. al, (2017) successfully deposited NiO_x_ with Cu as the hole transporting layer where the power conversion efficiency is about 20.5% with high conductivity and thermal stability [109]. In this study, the spin coating technique was carried out at 3000 rpm on the FTO surface for 40 s and the heat treatment was done for 1 h at 340 °C. On the other hand, Wei Chen et al., (2017) achieved 19.35% efficiency of PVSC with Cesium doped NiO_x_ hole transporting layer using the spin coating method. This study revealed the effectiveness of the Cs doped NiO_x_ layer for hole extraction of stable and effective inverted PVSC [110]. Moreover, the highest power conversion efficiency of 17.60% has been achieved for NiO_x_-based perovskite devices using the sputtering technique [111]. A 25 nm thick film was produced at room temperature with 1.97 W/cm^2^ power density and 0.24 Å/s deposition rate. The recent advancement of NiO_x_-based PSCs with the sputtering process is shown in Table 3. Huang et al., 2016 achieved 12.63% efficiency with NiO_x_ HTL PSCs using the co-sputtering method [127].

### 5.2. Cu_x_O

Cu_x_O is a conventional p-type semiconductor having high hole mobility and low cost due to its abundant reserves. Cu_x_O film can be deposited using a variety of simple techniques. It features a deep valence band, indicating a strong energy level matching with perovskite. It also possesses high hole mobility and a long lifetime of photo-excited carriers, making carrier transfer easier and reducing energy loss at the perovskite/HTL interlayer. Cu_x_O can be made with high quality using a variety of processes, including solution processing, sputtering, electrodeposition, vacuum evaporation, and so on, revealing its potential as an HTL material for efficient and long-lasting PSCs.

The highest power conversion efficiency of 19.0% was achieved by Rao et al., (2016) using Cu_x_O as the hole transporting layer of hybrid perovskite solar cell with a 75.8% of fill factor [133]. Chlorine was incorporated using a novel method called the one-step fast deposition-crystallization method in this study which improved the hole mobility and morphology of the film as well as the device recombination resistance. In 2018, the highest PCE of 18.85% was recorded using Cu_x_O as the HTL of PSCs with high stability [134]. After 500 h with 70–80% humidity in the open air, 30% of degradation has occurred. In this study, an organic–inorganic integrated HTL of FBT-Th_4_ polymer and Cu_x_O was deposited using the Vacuum thermal evaporation technique to reduce the heterogeneity. Table 4 shows the advancement of Cu_x_O-based HTL of PSCs for the last five years. Zuo and Ding [135] first used Cu_2_O and CuO as HTM in inverted planar PSCs. Cu_2_O was prepared by oxidizing CuI coatings on substrates to CuO in an aqueous NaOH solution. CuO is then produced by heating Cu_2_O and oxidizing it in the air. The devices based on Cu_2_O and CuO achieved enhanced PCEs of 13.35% and 12.16%, respectively, with a structure of ITO/HTL/MAPbI_3_/PC_61_BM/Ca/Al, which was superior to the devices based on PEDOT:PSS.

### 5.3. Other Metal Oxides Such as HTL

#### Graphene Oxide

Graphene and graphene-based oxide hole transporting materials are very much potential for PSCs for their decent electrical conductivity and excellent surface area compared to the other carbon-based HTMs. Therefore, the replacement of other hole transporting material, especially the organic hole transporting material by graphene oxide and reduced graphene oxide could be a good strategy to improve the oxide-based PSCs [47]. Graphene oxide-based perovskite solar cell was fabricated by Qing-Dan Yang et al., (2017) and achieved 16.5% power conversion efficiency without any hysteresis with the fill factor, V_OC_, and J_SC_ of 76.2%, 1.00, and 21.6 mAcm^−2^ respectively [143]. The higher stability of PSC was also recorded in this study and more than 80% PCE was recorded after 2000 h under moisture and successive light soaking condition. The PCE was dependent on thickness which is gradually and significantly decreased by increasing the film thickness from 2 nm to 10 nm. The power conversion efficiency of 15.1% was recorded by Li, Wenzhe, et al., (2014) with GO-based PSC [144]. Spin coating technique with 7000 rpm for 30 s was used to add the GO as the duel functional interface layer. The reduced graphene oxide-based HTM was used to develop highly efficient and stable PSCs by Leo et al., in 2015 [145]. In this research, they fabricate PSCs using a simple and room temperature process which showed the highest PCE of 10.8%. On the other hand, Palma et al., (2016) showed the highest stability of 2000 h with reduced graphene oxide-based PSCs [146]. The GO and rGO also act as a good additive with other hole transporting materials to enhance the efficiency as well as the stability of perovskite solar cells by improving the grain size and crystallinity of the materials [147]. Thus, by considering all the issues, the GO and rGO can be good candidates as bi-functional HTM to improve the performance of perovskite solar cells. Therefore, the study to optimize and improvement of GO particle size and film thickness still should be considered. The recent improvement of GO-based PSCs is listed below in Table 5.

### 5.4. CoO

The other normally used inorganic HTL is doped cobalt oxide which has been prepared by sputtering, in which doping is necessary for enhancing the conductivity and minimizing energy level mismatch with perovskite. Solar cells employing copper doped cobalt oxide (CoO_x_:Cu) prepared by reactive DC co-sputtering of cobalt and copper showed a decent efficiency of 9.89% [148]. Cu doping lifted the VBM of CoO_x_:Cu interlayer and removed the energy barrier due to the deeper VBM of CoO_x_ HTL than perovskite. Moreover, sputtering enabled fast deposition of the film with an optimal thickness of 10 nm, for the sake of high transmittance and low series resistance, within only 2 min and without the need to worry about the coverage issue when the sol-gel method was used.

The best photovoltaic performance of 14.5% with high stability was achieved from cobalt oxide-based PSC which was fabricated by Shalan Ahmed Esmail, et al., (2016) using a simple solution process [149]. The fabricated device showed more than 80% stability over 1000 h. Spinal structured cobaltite oxide (Co_3_O_4_) synthesized by the chemical precipitation method has been used as the hole extraction layer in carbon-based PSCs [150]. The film was formed by the skin printing method which enhances the performance and stability of the cell. It showed 13.27% efficiency with high stability of 2500 h under ambient conditions. Table 5 shows some latest studies on cobalt oxide HTL-based PSCs with their efficiency.

### 5.5. CrO

Chromium oxide-based hole transporting layer also enhances the hole extraction capacity of perovskite solar cells. A binary metal oxide heterojunction of copper and chromium was formed to fabricate a novel bimetal oxide-based PSC by Qin Ping-Li et al., (2017) which showed 17.19% efficiency on glass substrate and 15.53% efficiency on flexible PTE substrate [151]. The incorporated chromium oxide or metal-doped such as Cu doped chromium oxide plays a good role to improve the performance of Perovskite by developing the crystallinity, grain size, and surface properties of the Perovskite layer as well as the full cell [152,153].

**Table 5:** Recent advancement of other metal oxide HTL based perovskite solar cells.

Device Structure	Fabrication Processes	Efficiency (%)	FF	V_OC_	J_SC_	Year	References
ITO/GO/perovskite/C60/BCP/Au	Solution process	16.5	0.762	1.00	21.6	2017	[143]
FTO/PSK/GO	Spin coating	15.1	0.730	1.03	20.2	2014	[144]
ITO/(mixed with organic HTM)	Spin coating	11.90	0.705	0.88	19.18	2014	[154]
ITO/graphene oxide/PVK/PCBM/ZnO/Al	Spin coating	11.11	0.720	0.99	15.59	2014	[155]
ITO/reduced graphene oxide/PCBM/PCB/Ag	Spin coating	10.8	0.716	0.98	15.4	2015	[145]
CoO_x_/Glass/ITO/CoO_x_/Psk/PCBM/Ag	Solution process	14.5	0.755	0.949	20.28	2016	[149]
Co_3_O_4_ Glass/FTO/cl-TiO_2_/mp-TiO_2_/mp ZrO_2_/Psk/mp-Co3O4/carbon	Skin printing	13.27	0.64	0.88	23.43	2018	[150]
Co_1-y_Cu_y_O_x_/Glass/FTO/Co_1-y_Cu_y_O_x_/Psk/PCBM/Ag	Sputtering	9.98	0.599	0.925	17.98	2017	[149]
CH_3_NH_3_PbI_3_/Cu:CrO_x_	RF sputtering	14.76	0.71	1.03	20.17	2018	[153]
Cu:CrO_x_/Glass/FTO/Cu:CrO_x_/Psk/ PCBM/Ag	RF sputtering	10.99	0.7	0.98	16.02	2016	[152]
Cu_y_Cr_z_O_2_/Glass/FTO/Cu_y_Cr_z_O_2_/Psk/ PCBM/Ag	Solution process	15.3	0.7	1.07	20.48	2017	[151]

## 6. Inorganic HTL and Interface Engineering

The carrier dynamics, which are well recognized to be the driving force behind the photovoltaic performance of solar cells, should be carefully studied and regulated in order to minimize recombination losses and hence maximize device performance. Particularly, carrier dynamics in PSCs belong to different mechanisms, such as carrier dissociation, carrier transport, carrier extraction, carrier recombination, carrier accumulation (i.e., ionic and electronic carriers), and carrier collection. Among them, carrier extraction, recombination, accumulation, and collection are directly dependent on the quality of interfaces, suggesting that interfacial carrier dynamics is a key and plays a decisive role in final device performance.

Although the charge separation at the perovskite/HTL interface does not appear to be an issue, however, interfacial engineering is still obligatory to minimize recombination at the interface and to increase stability. These interface materials could be polymers, organic and inorganic materials. Particularly, hole migration from the interior perovskite to the surface may be encouraged by alteration of the perovskite/HTM contact. Interestingly, halide perovskite often exhibits excellent charge separation at the perovskite/HTL interface according to PL investigations. Lee et al., first reported the MAPbI_3_ as an interlayer between FAPbI_3_ and HTL [156]. However, a quick coating procedure with the high spinning rate of 6000 rpm was carried out to prevent the prepared FAPbI_3_ from completely dissolving during the spin-coating process for the second layer of MAPbI_3_. A very thin MAPbI_3_ layer was fabricated and an improved IPCE was observed. The mixed-cation perovskite was reported to use a similar strategy. In this case, FABr was spin-coated on top of mixed perovskite, (FAPbI_3_)0.85(MAPbBr_3_)0.15 film with the presence of PbI_2_ and a thin perovskite interlayer was created in the form of FAPbBr_3-x_ I_x_ as a consequence of reaction with the excess PbI_2_ present on the surface [157]. At the junction of perovskite and HTL, this wide bandgap passivation or interlayer serves as a barrier for charge carrier recombination. As a result, a considerable improvement in Voc is seen. Moreover, perovskite grain growth along with the compositional has been observed while spin-coated MABr solution on the MAPbI_3_ thin film [158]. The coating of MABr influences the passivation of the perovskite layer’s surface, improving photovoltaic stability and performance. In addition, Quantum dots (QDs) coated thin films have also been discovered to be prospective candidates for interfacial engineering materials for PSCs. A higher PCE is reported for MAPbI_3_ PSC with MAPbBr_3-x_I_x_ QDs interlayer than the PSC without QDs [159].

**Figure 11:**
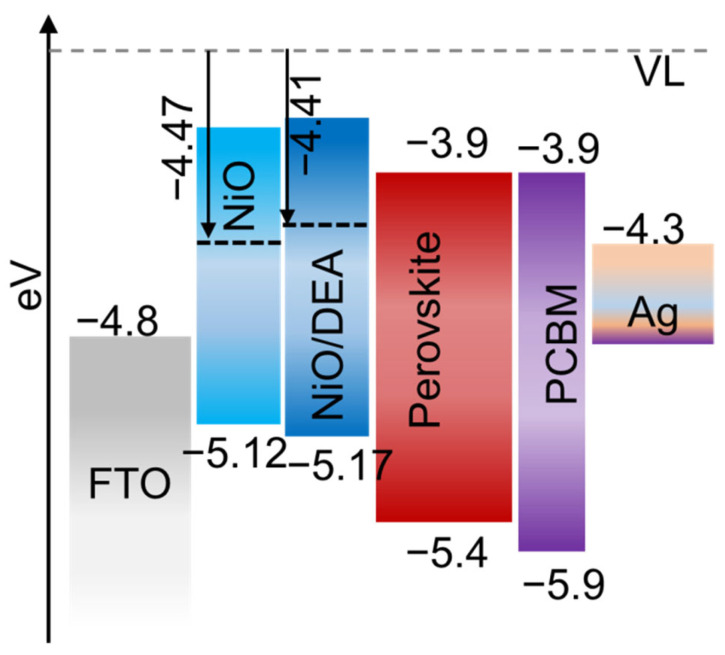
Schematic energy levels of each layer in the perovskite solar cell. (The work function (WF) of NiO was marginally reduced from 4.47 to 4.41 eV after DEA modification as reported [103]).

The hydrophobic and conductive polymers are also good candidates as interlayer materials. The improvement in Jsc and Voc was reported for using the poly-N-vinyl carbazole (PVK) as an interlayer at the perovskite/HTL interface [68]. Additionally, the inclusion of insulating poly(methyl methacrylate) (PMMA) between the interface of perovskite/spiro-MeOTAD was reported to be improved the photovoltaic performance, as well as the stability against moisture [160]. Particularly, the perovskite/spiro-MeOTAD interface has been studied more extensively by numerous researchers than any other materials and details of the studies could be found elsewhere [161]. Graphene oxide (GO) which particularly has high conductivity and ambient stability has also been used as an interlayer material in PSCs. The PEDOT:PSS-GO: NH3 bilayer was shown to better align the energetics between the ITO and valence band of the perovskite layer when GO:NH3 was utilized as an interlayer between PE-DOT:PSS and perovskite films [162]. A rise in photovoltaic performance was consequently seen. Similar results were obtained when mixed PEDOT:PSS and GeO2 were used, producing high WF and conductivity [163]. It should be noted that due to the higher WF, in the case of inverted PSCs, inorganic HTM NiO (Nickel Oxide) was observed to have a higher Voc than those based on PEDOT:PSS (&5.2 eV for NiO vs. & 5 eV for PEDOT:PSS) [164]. Furthermore, NiO is the most studied p-type HTM for the inverted PSCs compared to any other inorganic materials, however, the morphology and thickness of NiO significantly affect charge collection and recombination and cause an inconsistent photovoltaic performance. Figure 11 showed the effect of interlayers in PSCs.

NiO-based inverted structures have recently been found to have high PCEs reaching 22.13% [107]. Interface engineering is needed to further enhance charge carrier collection and decrease recombination at the interface. Particularly, the inferior quality of the perovskite layer formed on the NiO substrate and an improper interface is responsible for the relatively low PCEs from NiO-based PSCs as compared to conventional mesoscopic devices. As an interlayer modifier, diethanolamine (DEA) was utilized to improve the connectivity between the NiO-amine and perovskite-hydroxyl groups [103]. It has been reported that the WF of NiO is slightly decreased from 4.47 eV to 4.41 eV after the inclusion of DEA on NiO, as an indication of chemical interactions between NiO and DEA; however, Jsc and FF were significantly improved due to this surface modification. Moreover, the NiO layer with Au nanoparticle island interlayer showed higher EQE and thus higher Jsc than the bare NiO film as reported [165]. NiO_x_ NCs have also been implemented at the perovskite/Spiro-MeOAD interface and PCE was observed to increase from 18.51% to 19.89% [166]. Moreover, inorganic oxides Al2O3 [167,168,169], MgO [170] and Ta-WO_x_ [171] have been investigated as a perovskite/HTL interface material along with organometallic and organic materials (e.g., titanium acetylacetonate (TiAcac), zirconium acetylacetonate (ZrAcac), and hafnium acetylacetonate (HfAcac)) [114] and alternative to the organic interface materials [172,173,174,175].

**Figure 12:**
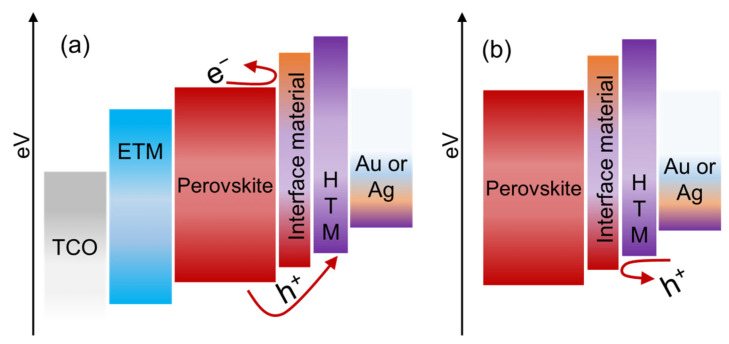
(**a**) Schematic of electron back transfer and (**b**) hole back transfer process.

Al_2_O_3_ has been utilized by several groups as an interface layer between TiO_2_/perovskite interfaces [167,168,169]. As the Al_2_O_3_ has a wide bandgap and a much higher CVM, it can suppress nonradiative recombination via blocking the electron back transfer as shown in Figure 12a, and improve the device efficiency. Additionally, using Al_2_O_3_ interface material, the cell UV and moisture stability have been observed to improve. After 70 days of exposure to humidity ranging from 40 to 70%, the unencapsulated device containing Al_2_O_3_ kept 60 to 70% of its initial PCE, but the control device without Al_2_O_3_ only retained 12% [176]. Other than the perovskite/HTL interface in normal PSCs, the thin Al_2_O_3_ layer was also implemented in the HTL/perovskite interface in inverted PSCs, and the improvement in PCE was achieved due to the blocking of back transfer of holes as shown in Figure 12b [177]. It is important to note that there are two ways to fabricate the Al_2_O_3_ interface layer: sol-gel or atomic layer deposition (ALD). The latter method is thought to be superior to the former due to its ability to control the film thickness.

It has been demonstrated that the performance and stability of devices are significantly influenced by the interface layer between the perovskite and HTM. In the not-too-distant future, it is hoped that we will have a perfect interface material that will guarantee the stability of PSCs within the anticipated level.

## 7. Efficiency and Stability Issues in Oxide-Based PSC

Alongside the efficiency of inorganic PSCs, stability is also a very important parameter that should be considered to designing a good PSC. The stability of PSCs depends on some the factors such as humidity, water, air, moisture, temperature, light, etc. Moreover, from this study, it is clear that the stability also depends on the HTMs used in the PSCs. Table 6 shows the stability of different inorganic HTM-based PSCs with their highest efficiency including different deposition techniques. From this study, it has been shown that the NiO_x_ showed more than 21% efficiency with high stability of 90% for more than 1200 h [91]. Spin coating deposition technique showed the highest performance whereas spray pyrolysis showed 90% stability over 500 h. On the other hand, CuO HTM-based PSC showed 90% stability over 500 h with the highest efficiency of 18.85% using the thermal evaporation technique [133]. The stability of PSCs mostly depends on the stability of perovskite and HTMs; therefore, it is a big concern to the researchers to develop such type of HTMs which enhance the solar cell lifetime with a low-cost fabrication technique. The development of inorganic materials with hydrophobic properties can be a good option to maximize the cost-effective as well as moisture, air, and thermally stable PSCs. Generally, the inorganic HTMs are less responsive to UV, humidity, moisture, and temperature compared the organic HTMs. Furthermore, the binding energy of inorganic materials is large with fewer free electrons and it may act as the protective layer of PSC. Among all deposition techniques, Spin coating showed the highest performance in accounts of efficiency and stability [107]. However, it is difficult to compare one technique with another because some techniques are physical and some are chemical. Among the chemical process, they are divided into two groups, such as gas phase and the solution process. Furthermore, the processes are depending on the materials used in it. However, the efficiency and stability of the IHTM-based PSCs could be improved by taking some strategies besides the synthesis of IHTM and fabrication techniques. Some important strategies are solvent engineering, interfacial engineering, bandgap engineering, bandgap adjustment, increasing the charge generation, and the enlargement of perovskite’s grain size [178].

## 8. Future Outlook

**Figure 13:**
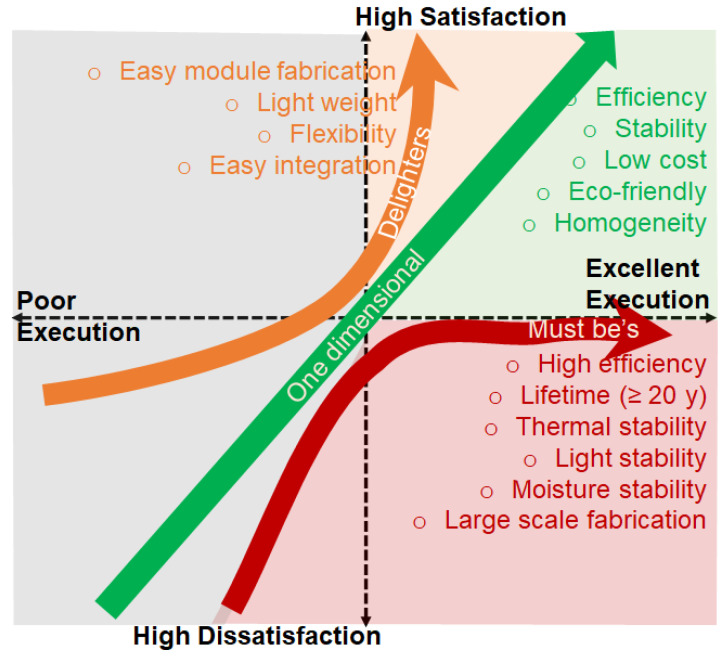
Proposed Kano model for successful commercialization.

One aspect of the PSCs that is currently attracting strong attention is their high PCE, low cost, and easy fabrication technique. Although they are an intrinsic advantage of PSC-PV technology; however, to move these new solar cells beyond the lab, an upscale in size and long-term stability is necessary. Although above 25% efficiency has already been achieved, the lifespan is, at most, a few thousand hours only and is still insufficient for practical implementation. Certainly, the stability of PSCs should extend more than 10 years for commercialization including prioritizing the new product features. It should be mentioned that entering a niche market with creative services or distinctive product qualities that are unreachable by the best alternative (e.g., silicon photovoltaics) is a vital stage in commercializing a breakthrough technology. Thus, prioritizing the PSCs’ features such as high efficiency, high stability, and large-scale production is expected to grow over time for cutting specific costs. Furthermore, R&D must identify and target niches to produce market pull to advance the advent of any new technology by either generating new markets or displacing a mature technology in current markets. Kano model as assumed in Figure 10 could help us in developing perovskite solar cells for prioritizing in the market [151,179]. Particularly, the Kano model is distinguished by its rigorous focus on customer perception, and existing and/or possible new product features are classified based on how much satisfaction they may provide. In Figure 13 we proposed such classified features of PSCs required for commercial success beyond the current market of solar cells. The performance and value of the product are linearly related to the one-dimensional requirements: the better they are completed, the more satisfaction they bring, and vice versa. The must be’s are the technology’s fundamental expectations and the minimum conditions for market access. Strong research efforts in these areas are essential for market penetration. Customers are surprised and delighted by delighters, which provide innovation and extra value. The delighters are especially applicable when the market of a specific technology becomes mature.

As previously stated, while PSCs have already attained better efficiencies than the commercially dominant c-Si solar cells, stability, large-scale production, and cost minimization remain a challenge. It should be noted that several aspects such as modification of the structural design, use of different charge and electron transport materials including different metal-oxide films, different hole transport materials which have hydrophobic nature, modification in the electrode material preparation and encapsulation procedures, have already been considered by the various researchers for systematic engineering of perovskites to improve their stability. It has been observed that the current modification in perovskite materials, device structure, and/or interface material is not enough for achieving higher stability, and we recommend developing new materials and designs that may play a great role to overcome the aforementioned issues in the perovskite PV field. Besides this, the stability data provided by various researchers can’t be fairly compared because different experiment uses different testing conditions, such as humidity, temperature, and encapsulation technique. Particularly, PCE is a well-defined parameter that can be validated according to standards, whereas stability-related characteristics like lifetime and deterioration rates cannot. Thus, it is crucial need to standardize the required test condition for stability testing of PSCs focusing on mechanical stability, thermal stability, device hysteresis, and stability under exposure to light, moisture, and oxygen for each fabrication technique.

Even though PSCs seem to have extremely low manufacturing costs due to solution processing-based fabrication and found a strong alternative photovoltaic technology to compete with silicon, it is very difficult for PSCs as new technology to enter the commercial market due to the high capital costs of established photovoltaics manufacturing. The fabrication cost of PSCs could be further reduced via structural modification, such as omitting HTL and/or ETL. Moreover, using carbon (C) electrodes instead of metal electrode could ease the fabrication process and reduce the cost as well. Particularly, fabricating metallic electrodes requires a high vacuum and costly instrument whereas, a C electrode can be coated by a simple doctor’s blade or screen-printing method. Numerous researchers have used carbon nanotubes/carbon paste/carbon black as counter electrodes and achieved noticeable PCE [180,181,182], however more research is needed to increase the stability and efficiency.

On the other hand, Lead (Pb) is a toxic element that is a barrier to the commercialization of PSC. Researchers are attempting to replace Pb with other components that have suitable properties for making perovskites a good photon absorber material. Numerous alternative materials, such as Sn [183], Sb [184], and Bi [185], have already been explored; however, their efficiency and stability are not yet found as high as Pb-based PSCs. Finally, we would like to propose two recommendations for achieving higher stability towards future commercialization of PSCs: (i) stability tests of PSCs should be conducted using the standard protocol, such as ISOS. This will allow for a more accurate comparison of materials and device architectures, as well as a clearer picture of the research path toward extended lifetime, and (ii) the modification of fabrication technique as well as device structure and materials should comply with the ease of large-scale PSC fabrication. Moreover, new materials and designs with high stability under harsh conditions are also desirable to improve the performance of PSCs. The new materials can be used in a variety of ways, from improving existing materials to building new ones.

## 9. Conclusions

In the present review work, the progress in perovskite solar cells using inorganic oxide-based HTMs towards high efficiency and high stability has been discussed comprehensively. Perovskite materials have already been proven to be most suitable for solar energy harvesting, owing to their tuneable band gap, high absorption coefficient, and long diffusion length. Due to these excellent properties, the efficiency of PSCs increased from 3.8% to 25.8% in a short period. One of the major issues in the commercialization of PSC is its instability in the presence of air, and another is the presence of toxic lead. Particularly, the soft and ionic nature of perovskite materials makes it more challenging to acquire the long-time stability of PSCs. CH_3_NH_3_PbI_3_ is thermally unstable during perovskite layer deposition and quickly degrades to PbI_2_. As a result, the mechanism of CH_3_NH_3_PbI_3_ degradation will need to be investigated further in the future. Moreover, several aspects have been considered by various researchers for systematic engineering of PSCs to improve their stability, including changing the structural design, using different ETLs and HTLs, including different metal-oxide films with hydrophobic nature, and changing the electrode material preparation and encapsulation procedures. Furthermore, many researchers are attempting to replace Pb with Sn/Ge, with encouraging results. In the future, researchers might look into how to improve the stability of PSC in various settings. We hope that PSCs’ perspective will encourage the PV community to focus on the areas that are most important to its commercial success, such as (i) continuing to focus on active materials with an emphasis on environmental stability and non-toxic elements without sacrificing high PCE; (ii) increased focus on device light and moisture stability, as well as field investigations on device degradation modes; (iii) more devotion to module design; and (iv) enhancements to the roll to roll processable fabrication technique. Perovskite, as an emerging PV technology, must supplement or expand the market’s existing capabilities for successful commercialization. We firmly believe that the future is bright due to the unified focus on PSCs and their extraordinarily quick advancement in recent years. Hence, this paper will be extremely valuable to newcomers to the field of PSC research who are looking for a thorough examination of the current state of perovskite solar cells.

## Figures and Tables

**Table 1:** List of recently published review articles on inorganic HTMs for PSCs.

No.	Title	Journal	Year	References
01	Recent progress of inorganic hole transport materials for efficient and stable perovskite solar cells	Nano Select	2021	[41]
02	A brief review of hole transporting materials commonly used in perovskite solar cells	Rare Metals	2021	[42]
03	Nickel Oxide for Perovskite Photovoltaic Cells	Advanced Photonics Research	2021	[43]
04	Toward efficient and stable operation of perovskite solar cells: Impact of sputtered metal oxide interlayers	Nano Select	2021	[44]
05	Inorganic hole transport layers in inverted perovskite solar cells: A review	Nano Select	2021	[45]
06	Progress, highlights, and perspectives on NiO in perovskite photovoltaics	Chemical Science	2020	[46]
07	A review on the classification of organic/inorganic/carbonaceous hole-transporting materials for perovskite solar cell application	Arabian Journal of Chemistry	2020	[47]
08	Review of current progress in inorganic hole-transport materials for perovskite solar cells	Applied Materials Today	2019	[48]
09	Recent progress of inorganic perovskite solar cells	Energy & Environmental Science	2019	[49]
10	Inorganic hole transporting materials for stable and high efficiency perovskite solar cells	The Journal of Physical Chemistry C	2018	[50]
11	Analysing the prospects of perovskite solar cells within the purview of recent scientific advancements	Crystals	2018	[51]
12	Recent progress in stability of perovskite solar cells	Journal of Semiconductors	2017	[52]
13	Emerging of inorganic hole transporting materials for perovskite solar cells	The Chemical Record	2017	[53]
14	Recent advances in the inverted planar structure of perovskite solar cells	Accounts of chemical research	2016	[54]
15	The progress of interface design in perovskite-based solar cells	Advanced Energy Materials	2016	[55]
16	Recent progress on hole-transporting materials for emerging organometal halide perovskite solar cells	Advanced Energy Materials	2015	[36]

**Table 2:** Hole mobility and price of some representative inorganic HTMs along with organic HTMs (spiro-MeOTAD, PTPAA, and PEDOT:PSS) (note: the price is arbitrary).

HTMs	Mobility (cm^2^/V-S)	Price (per Gram, USD)	Reference
Spiro-OMeTAD	4 × 10^−5^	422	[82]
PTAA:poly(triarylamine)	10^−2^–10^−3^	1145	[83]
PEDOT:PSS	1 × 10^−2^	166	[84]
NiO_x_	0.14	14	[85]
V_2_O_5_	0.23	49	[86]
MoO_3_	0.4	22	[32]
WO_3_	0.25	15.6	[87]
Cu_2_O	100	2.96	[53]
CuO	0.129	2.96	[53]
CuSCN	0.01–0.1	3	[53]
Cu_2_ZnSnS_4_	6.0–30	-	[53]
CuAlO_2_	3.6	5.16	[88]
CuCrO_2_	7.6	22.4	[89]
CuGaO_2_	0.01–10	3.49	[30]

**Table 3:** The efficiency of NiO_x_ base HTL in PSCs using different deposition techniques.

Device Structure	Deposition Technique	Efficiency (%)	FF	V_OC_ [V]	J_OC_ (J-V) [mAcm^−2^]	Year	References
NiO_x_/F_2_HCNQ	Spin coating	22.13	0.82	1.14	23.44	2020	[107]
ITO/SnO_2_/(FAPbI_3_)_x_(MAPbBr_3_)_1−x_/NiO_x_/spiro-OMeTAD/Au	Spin coating	21.66	0.79	1.14	23.82	2020	[106]
ITO/NiO_x_/MA_1-y_FA_y_PbI_3-x_Cl_x_/2D-3D perovskite/PCBM/BCP/Ag	Spin coating	21.4	0.83	1.12	23.1	2019	[108]
FTO/NiO_x_/PVK/PCBM/ZrAcac/Al	Spin coating	20.5	0.77	1.12	23.07	2017	[109]
ITO/NiO_x_/MSs/perovskite/PC_61_BM/BCP/Ag	-	20.34	0.80	1.12	22.34	2021	[112]
NiO/TSPA(p-i-n)	Spin coating	20.21	-	-	-	2021	[113]
FTO/Cs:NiO_x_/PVK/PCBM/ZrAcac/Ag	Spin coating	19.35	0.79	1.12	21.77	2017	[110]
p-i-n	Spin coated	19.0	0.77	1.05	23.17	2019	[92]
ITO/NiO_x_/PVK/PCBM/ZrAcac/Al	Spin coating	18.69	0.78	1.079	22.17	2017	[114]
ITO/Cu:NiO_x_/PVK/PCBM/BCP/Ag	Spin coating	18.66	0.81	1.11	20.76	2017	[115]
FTO/NiO_x_/PVK/PCBM/Ag	Spin coating	18.6	0.75	1.09	22.8	2017	[116]
ITO/NiO_x_/Psk/PCBM/Au	Spin coating	18.23	0.47	0.79	6.4	2016	[117]
ITO/NiO_x_/PVK/PCBM/c-HATNA/Bis-C_60_/Ag	Spin coating	18.21	0.79	1.09	21.25	2018	[118]
FTO/cp-TiO_2_/mp-TiO_2_/mp-NiO/Psk/carbon (n-i-p)	Spin coating	18.2	0.71	0.89	11.4	2016	[119]
ITO/NiO_x_/Psk/PCBM/C_60_/BCP/Al	Spin coating	18	0.56	1.06	10.6	2016	[100]
ITO/NiO_x_/PVK/PCBM/Al	Spin coating	18.0	0.74	1.12	21.79	2018	[120]
ITO/NiO_x_/PVK/PCBM/Ag	Spin coating	17.2	0.78	1.03	21.4	2017	[121]
ITO/NiO_x_/PVK/PCBM/Ag	Spin coating	16.55	0.75	1.04	21.22	2017	[122]
ITO/NiO_x_/Psk/PCBM/Ag	Spin coating	16.47	0.75	1.07	20.58	2016	[123]
-	Spin coating	16.4	0.67	1.12	21.8	2020	[123]
ITO/NiO_x_/PVK/ZnO/Al	Spin coating	16.1	0.76	1.01	21.01	2016	[119]
ITO/NiO_x_/PVK/PCBM/LiF/Al	Spin coating	13.4	0.69	1.03	19	2016	[124]
ITO/NiO_x_/CH_3_NH_3_PbI_3_/PCBM_60_/ZnO NPs/BCP/Al	Spin coating	13	0.61	1.03	21	2021	[125]
FTO/cp-TiO_2_/mp-TiO_2_/mp-NiO/Psk/carbon	Spin coating	11.4	0.71	0.89	18.2	2016	[126]
ITO/NiO_x_/MAPbI_3_/PCBM/BCP/Ag	Sputtering	17.6	0.79	1.07	20.65	2018	[111]
FTO/Co:NiO_x_/MAPbI_3_/PCBM/Ag	Sputtering	12.63	0.63	1.01	20.02	2016	[127]
ITO/Li and Co:NiO_x_/MA_1-y_FAyPbI_3-x_Cl_x_/PCBM/BCP/Ag	Solution combustion process	20.1	0.78	1.09	23.8	2019	[96]
ITO/NiO_x_/CsBr/MA_1-y_FA_y_PbI_3-x_ Cl_x_/PCBM/BCP/Ag	Solution combustion process	19.7	0.75	1.09	23.5	2020	[91]
FTO/Sr:NiO_x_/MAPbI_3_/PCBM/BCP/Ag	Solution process	19.49	0.75	1.14	22.66	2019	[97]
FTO/K:NiO_x_/MAPbI_x_Br_3-x_/PCBM:C60/BCP/Ag	Solution process	18.05	0.78	1.01	22.77	2019	[98]
ITO/PLD-NiO_x_/Psk/PCBM/LiF/Al	e-beam evaporation	17.3	0.81	1.06	20.2	2015	[128]
Planar p-i-n FTO/NiO_x_/FAPbI3/PCBM/TiO_x_/Ag	Spraying	20.65	0.81	1.10	23.09	2017	[85]
FTO/NiO_x_/PVK/PCBM/Ag	Spray pyrolysis	19.58	0.77	1.12	22.68	2017	[129]
ITO/NiO_x_/Cs_0.05_MA_0.95_PbI_3_/PCBM/ BCP/AZO/Ag	Atomic layer deposition	18.4	0.78	1.05	22.56	2018	[99]
ITO/NiO_x_/CsMAFAPbI_3-x_Br_x_/C_60_/BCP/Cu	Atomic layer Deposition	17.07	0.73	1.07	21.75	2019	[130]
ITO/NiO_x_/MAPbI_3_/PCBM/Ag	Atomic layer deposition	16.4	0.72	1.04	21.9	2016	[131]
ITO/NiO_x_/PVK/PCBM/BCP/Ag	Vacuum deposition	15.4	0.78	1.06	18.6	2018	[132]
Planar p-i-n ITO/NiO_x_/PVK/PCBM/C_60_/BCP/Al	Vacuum thermal evaporation	10.6	0.56	1.06	18	2016	[100]
ITO/NiO_x_/PVK/PCBM/Ag	Electrodeposition	17.1	0.72	1.05	22.6	2017	[101]
Mesoscopic n-i-p FTO/c-TiO_2_/m-TiO_2_/PVK:NiO-MWCNTs	Drop casting	15.38	0.76	0.91	22.38	2017	[102]

**Table 4:** Recent advancement of CuO_x_ based perovskite solar cells.

Device Structure	Fabrication Processes	Efficiency (%)	FF	V_OC_	J_SC_	Year	References
ITO/CuO_x_/Psk/PCBM/C_60_/ BCP/Ag	Spin coating	19.0	0.758	1.11	22.5	2016	[133]
FTO/SnO_2_/ PCBM/MAPbI_3_/FBT-Th_4_/Cu_x_O/Au	Thermal evaporation	18.85	0.75	1.12	22.35	2018	[134]
ITO/CuO_x_/Psk/PC_61_BM/ZnO/Al	Vapor deposition	17.43	0.76	1.03	22.42	2017	[136]
ITO/CuO_x_/Psk/C_60_/BCP/Ag	Spin coating	17.1	0.744	0.99	23.2	2016	[28]
ITO/Cu_2_O/Psk/PCBM/Ag	Sputtering	11.03	0.662	0.95	17.5	2016	[137]
ITO/Cu_2_O/Psk/C_60_/Bphen/Ag	Electrodeposition	9.64	0.61	0.88	18.03	2016	[138]
FTO/TiO_2_/Psk/Cu_2_O/Au	Sputtering	8.93	0.59	0.96	15.8	2016	[139]
ITO/Cu_2_O/Psk/PCBM/Al	SILAR	8.23	0.56	0.89	16.52	2016	[140]
ITO/CuO–Cu_2_O/Psk/C_60_/BCP/Ag	Sputtering	8.1	0.586	0.96	14.4	2016	[141]
ITO/CuO_x_/Psk/C_60_/BCP/Al	Electrospray	5.83	0.48	0.7	17.22	2017	[142]
ITO/CuO_x_/Psk/PCBM/C_60_/BCP/Ag	Spin coating	19.0	0.758	1.11	22.5	2016	[133]

**Table 6:** Stability chart of different inorganic HTM-based PSCs.

IHTMs	Deposition Technique	Highest Efficiency (%)	Highest Stability	References
NiO_x_	Spin-coating	21.66	90% over 1200 h	[91]
	Sputtering	17.6	-	[111]
	Spray pyrolysis	20.65	90% over 500 h	[91]
	Solution combustion process	20.1	-	[96]
	Atomic layer deposition	18.4	86.7% over 500 h	[99]
	Others	17.1	-	[101]
CuO	Spin-coating	19.0	-	[133]
	Sputtering	11.03	40% over 500 h	[137]
	Vapor deposition	17.43	90% over 500 h	[136]
	Thermal evaporation	18.85	90% over 500 h	[133]
	Electrodeposition	9.64	-	[138]
Graphene oxide	Solution process	16.5	80% over 20,000 h	[143]
CoO	Solution process	145	80% over 1000 h	[149]
	Skin printing	13.27	2500 h	[150]
CrO	Solution process	15.3	-	[151]

## Data Availability

Not applicable.
